# Association between carotid atheroma and cerebral cortex structure at age 73 years

**DOI:** 10.1002/ana.25324

**Published:** 2018-10-05

**Authors:** Saud Alhusaini, Sherif Karama, Tuong‐Vi Nguyen, Alexander Thiel, Boris C. Bernhardt, Simon R. Cox, Janie Corley, Adele Taylor, Alan C. Evans, John M. Star, Mark E. Bastin, Joanna M. Wardlaw, Ian J. Deary, Simon Ducharme

**Affiliations:** ^1^ Department of Neurology and Neurosurgery, Montreal Neurological Institute and Hospital McGill University Montreal Quebec Canada; ^2^ McConnell Brain Imaging Centre, Montreal Neurological Institute McGill University Montreal Quebec Canada; ^3^ Department of Psychiatry, Douglas Mental Health University Institute McGill University Montreal Quebec Canada; ^4^ Department of Psychiatry, McGill University Health Centre McGill University Montreal Quebec Canada; ^5^ Department of Obstetrics–Gynecology, McGill University Health Centre McGill University Montreal Quebec Canada; ^6^ Department of Neurology Jewish General Hospital, Lady Davis Institute for Medical Research Montreal Quebec Canada; ^7^ Centre for Cognitive Ageing and Cognitive Epidemiology, Department of Psychology University of Edinburgh Edinburgh United Kingdom; ^8^ Alzheimer Scotland Dementia Research Centre, Department of Psychology University of Edinburgh Edinburgh United Kingdom; ^9^ Brain Research Imaging Centre, Centre for Clinical Brain Sciences University of Edinburgh Edinburgh United Kingdom; ^10^ UK Dementia Research Institute at the University of Edinburgh Edinburgh United Kingdom

## Abstract

**Objective:**

To examine the relationship between carotid atherosclerosis and cerebral cortical thickness and investigate whether cortical thickness mediates the association between carotid atheroma and relative cognitive decline.

**Methods:**

We assessed 554 community‐dwelling subjects (male/female: 296/258) from the Lothian Birth Cohort 1936 who underwent brain magnetic resonance imaging and carotid Doppler ultrasound studies at age 73 years. The relationship between carotid atherosclerosis markers (internal carotid artery stenosis, intima–media thickness, velocity, pulsatility, and resistivity indexes) and vertex‐wide cerebral cortical thickness was examined cross‐sectionally, controlling for gender, extensive vascular risk factors (VRFs), and intelligence quotient at age 11 (IQ‐11). We also determined the association between carotid stenosis and a composite measure of fluid intelligence at age 73 years. A mediation model was applied to examine whether cortical thickness mediated the relationship between carotid stenosis and cognitive function.

**Results:**

A widespread negative association was identified between carotid stenosis (median = 15%) and cerebral cortical thickness at age 73 years, independent of the side of carotid stenosis, other carotid measures, VRFs, and IQ‐11. This association increased in an almost dose–response relationship from mild to severe degrees of carotid stenosis, across the anterior and posterior circulation territories. A negative association was also noted between carotid stenosis and fluid intelligence (standardized beta coefficient = −0.151, *p* = 0.001), which appeared partly (approximately 22%) mediated by carotid stenosis‐related thinning of the cerebral cortex.

**Interpretation:**

The findings suggest that carotid stenosis represents a marker of processes that accelerate aging of the cerebral cortex and cognition that is in part independent of measurable VRFs. Cortical thinning within the anterior and posterior circulation territories partially mediated the relationship between carotid atheroma and fluid intelligence. Ann Neurol 2018;84:576–587

Carotid atherosclerosis is a significant risk factor for stroke and has been associated with cognitive decline and dementia.^1^, ^2^ Vascular risk factors (VRFs) account for about 65% of the variance in large artery atherosclerosis, including carotid disease, which often manifests as increased carotid intima–media thickness (CIMT), carotid plaque, and vessel stenosis.^3^, ^4^

Carotid atherosclerosis markers were previously linked to altered brain structure and silent cerebral infarction in asymptomatic individuals.^5^ Increased CIMT and carotid stenosis ≥ 25% were found to correlate with lower total brain volume and larger white matter hyperintensities (WMHs).^5^ Similarly, in symptomatic patients with evidence of large‐artery atherosclerosis, increased CIMT and carotid stenosis were linked to decreased total brain and gray matter (GM) volumes.^6^ Such global brain atrophy appeared progressive in individuals with high‐grade carotid stenosis (≥50%).^6^ A negative correlation between CIMT and the thickness of the parietal cortex was also reported in community‐based older individuals after controlling for multiple VRFs.^7^ These observations suggest that carotid atherosclerosis is associated with alteration in GM structure. However, the extent and anatomical distribution of this association between GM changes and carotid atherosclerosis markers is not fully understood. Furthermore, it is unclear whether these associations are restricted to individuals with clinically significant carotid disease (eg, those with high‐grade carotid stenosis), and whether they are causative, related to other VRFs, or represent a marker of another vascular dysfunction, such as arterial wall stiffness.

The relationship between carotid atherosclerosis and cognitive function has also previously been investigated. Lower scores on processing speed and executive functioning tasks were reported in older and middle‐aged individuals with increased CIMT.^8^, ^9^, ^10^, ^11^ In a recent study of the Lothian Birth Cohort 1936 (LBC1936; also used in this study), increased arterial stiffness, as indicated by the pulsatility and resistivity indices measured in the internal carotid arteries, but not carotid stenosis, correlated significantly with slower processing speed and worse visuospatial function at age 70 years, and declining crystallized intelligence over a 6‐year follow‐up period.^12^ It is not known whether the correlations between carotid atherosclerosis and cognitive function performances are mediated by carotid disease–related thinning of cortical thickness, or whether they reflect shared coassociation with VRFs and age.

In the present study, we examined the relationship between cerebral cortical thickness and multiple markers of carotid atherosclerosis cross‐sectionally at age 73 years in a large sample of community‐dwelling older individuals, the LBC1936. We hypothesized that carotid atherosclerosis markers would be associated with thinner cerebral cortex and expected the thinning in cerebral cortical thickness to partially mediate any relationship between carotid atherosclerosis and cognitive function.

## Subjects and Methods

### *Subjects*

**Figure 1:**
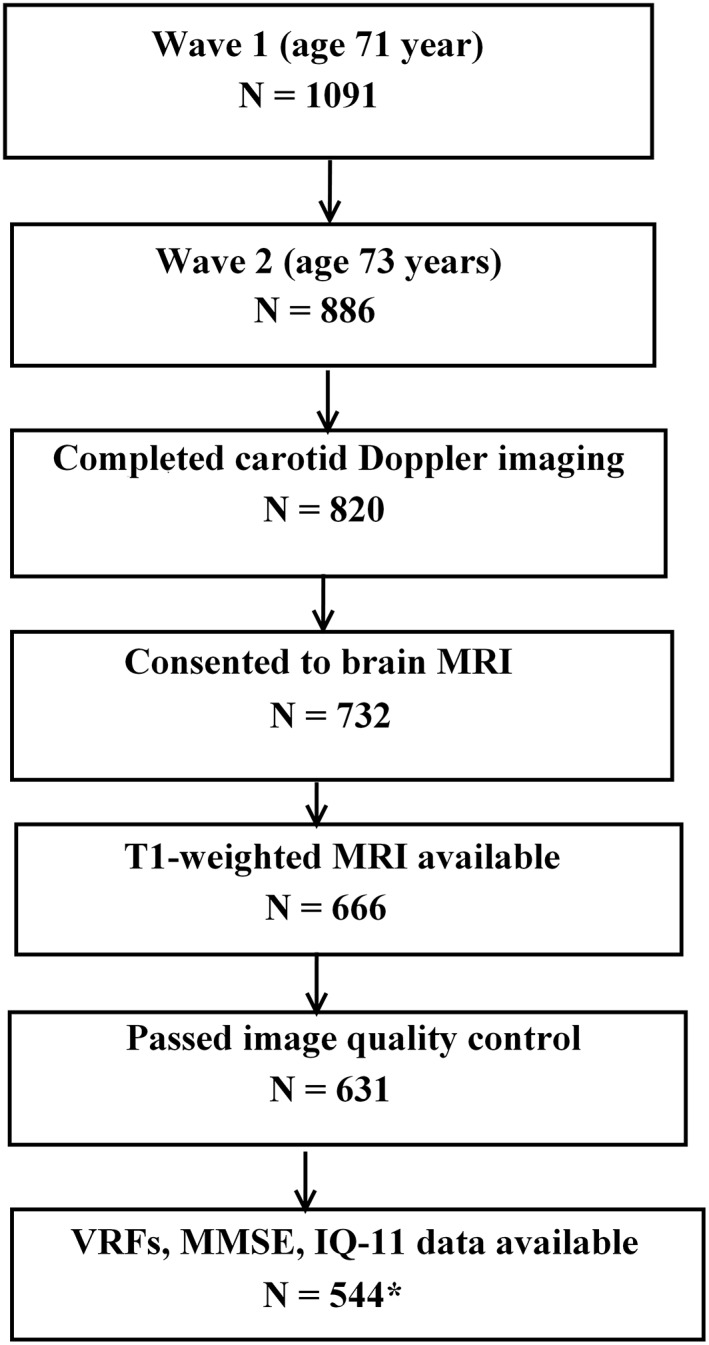
Flowchart indicating the selection process of the Lothian Birth Cohort 1936 subjects included in the final sample for data analyses. *Final sample included in the cross‐sectional analyses reported here. IQ‐11 = intelligence quotient at 11 years; MMSE = Mini‐Mental State Examination; MRI = magnetic resonance imaging; VRF = vascular risk factor.

This study was performed using data from the LBC1936.^13^ Participants in the LBC1936 study had mostly taken part in the Scottish Mental Survey of 1947, in which almost all Scottish school children born in 1936 and attending school in 1947 took the same general cognitive ability test at age 11 years. A total of 1,091 community‐dwelling individuals took part in the first wave of the LBC1936 study between 2004 and 2007, when they were about 70 years old. In the first wave, each participant completed a set of cognitive and medical tests, including a detailed physical and health evaluation. The second wave of the study took place between 2007 and 2010, when participants were aged about 73 years. During the second wave, subjects were invited to undergo brain magnetic resonance imaging (MRI), carotid artery Doppler ultrasound scanning, and repeated cognitive and medical testing.^14^ From the 866 individuals who participated in the second wave, carotid Doppler imaging was completed in 820 subjects. A combination of high‐resolution structural T1‐weighted brain MRI and Doppler ultrasound studies of the carotid arteries was available for 666 individuals. Of those, 631 passed image quality control for cortical thickness analysis. Data on all relevant confounding variables (detailed below) were available for 554 individuals who were included in the final analyses of the current study (Fig 1). A detailed description of study participants is presented in Table 1.

**Table 1:** Characteristics and Clinical Variables of Study Participants at Age 73 Years

Clinical Variable	All Sample	0% Carotid Stenosis	1–24% Carotid Stenosis	25–49% Carotid Stenosis	≥50 Carotid Stenosis
Total, n (%)	554	83 (15%)	286 (51.6%)	161 (29.1%)	24 (4.3%)
Gender, n (%)					
Male	296 (53.4%)	44 (53.0%)	153 (53.5%)	86 (53.4%)	13 (54.2%)
Female	258 (46.6%)	39 (47.0%)	133 (46.5%)	75 (46.6%)	11 (45.8%)
Age, yr, mean (SD)	72.7 (0.73)	72.5 (0.75)	72.8 (0.72)	72.8 (0.70)	72.5 (0.81)
IQ‐11, mean (SD)	101.6 (15.3)	104.0 (12.7)	102.0 (15.3)	99.9 (16.6)	100.5 (14.2)
MMSE, mean (SD)	28.8 (1.3)	29.1 (1.0)	28.9 (1.2)	28.6 (1.4)	29.3 (1.0)
Carotid variables, mean (SD)					
Carotid stenosis, %	12.45	0	7.2 (5.1)	25.4 (6.9)	44.3 (10.5)
Velocity, m/s	32.67 (10.7)	31.3 (7.8)	30.4 (6.8)	33.2 (9.0)	57.3 (23.9)
Pulsatility index	1.27 (0.23)	1.23 (0.24)	1.28 (0.22)	1.29 (0.23)	1.27 (0.21)
Resistivity index	0.68 (0.07)	0.66 (0.1)	0.68 (0.07)	0.69 (0.06)	0.68 (0.06)
CIMT	0.84 (0.17)	0.80 (0.14)	0.82 (0.16)	0.87 (0.19)	0.99 (0.24)
Fluid intelligence, mean (SD)					
Matrix Reasoning	13.6 (4.9)	13.0 (3.8)	13.5 (4.9)	13.3 (5.1)	13.0 (3.8)
Block Design	34.7 (9.7)	36.1 (10.6)	35.0 (10.0)	34.3 (9.0)	30.6 (6.5)
Digit Span Backward	7.9 (2.0)	8.5 (2.3)	8.0 (2.1)	7.5 (2.2)	7.5 (2.0)
Letter‐Number Sequencing	11.0 (2.9)	11.4 (3.1)	10.9 (2.9)	11.1 (3.0)	10.9 (2.7)
Digit Symbol	56.6 (11.6)	60.0 (10.3)	57.4 (11.8)	53.8 (11.3)	53.6 (12.0)
Symbol Search	24.9 (5.9)	26.3 (5.1)	25.2 (5.7)	23.7 (6.5)	23.7 (6.6)
VRFs, n (%)					
Hypertension					
Yes	262 (47.3%)	33 (39.8%)	141 (49.3%)	77 (47.8%)	11 (45.8%)
No	292 (52.7%)	50 (60.2%)	145 (50.7%)	84 (52.2%)	13 (54.1%)
Diabetes mellitus					
Yes	56 (10.1%)	8 (9.6%)	30 (10.5%)	17 (10.6%)	1 (4.2%)
No	498 (89.9%)	75 (90.4%)	256 (89.5%)	144 (89.4%)	23 (95.8%)
Hypercholesteremia					
Yes	229 (41.3%)	32 (38.6%)	116 (40.6%)	66 (41%)	15 (62.5%)
No	325 (58.7%)	51 (61.4%)	170 (59.4%)	95 (59.0%)	9 (37.5%)
Smoking					
Yes	42 (7.6%)	3 (3.6%)	24 (8.4%)	12 (7.5%)	3 (12.5%)
Ex‐smoker	241 (43.5%)	34 (40.0%)	123 (43.0%)	72 (44.7%)	12 (50.0%)
No	271 (48.9%)	46 (55.4%)	139 (48.6%)	77 (47.8%)	9 (37.5%)
BMI, mean (SD)	27.8 (4.4)	28.3 (5.0)	27.65 (4.1)	27.9 (4.8)	27.2 (3.9)
History of stroke, n (%)					
Yes	33 (6%)	3 (3.6%)	16 (5.6%)	12 (7.5%)	2 (8.3%)
No	521 (94%)	80 (96.4%)	270 (94.4%)	149 (92.5%)	22 (91.7%)
History of cardiovascular disease, n (%)					
Yes	146 (26.4%)	16 (19.3%)	77 (27.0%)	48 (29.8%)	5 (20.8%)
No	408 (73.6%)	67 (80.7%)	209 (73.0%)	113 (70.2%)	19 (79.2%)

BMI = body mass index; CIMT = carotid intima–media thickness; IQ‐11 = intelligence quotient at 11 years; MMSE = Mini‐Mental State Examination; SD = standard deviation; VRF = vascular risk factor.

Ethical approval for the LBC 1936 study was obtained from the Multi‐Centre Research Ethics Committee for Scotland and Lothian Research Ethics Committee. Written informed consent was obtained from all participants.

### *Clinical Assessments*

During a detailed clinical evaluation that included a medical history and physiological measures, the following VRFs were recorded: history of hypertension, hypercholesterolemia, diabetes mellitus, and smoking (current vs stopped > 1 year ago or never smoked).^13^, ^14^ History of cardiovascular disease, which included self‐reported incidents of coronary artery disease, stroke, peripheral vascular disease, and aortic disease, was noted. Systolic and diastolic blood pressures (an average of 3 sitting and standing measures) were measured.

### *Cognitive Assessments*

A cognitive test battery covering 4 domains of cognitive ability, including processing speed, visuospatial ability, memory, and crystallized intelligence, was completed.^15^, ^16^

Visuospatial ability was measured by Matrix Reasoning and Block Design from the Wechsler Adult Intelligence Scale, 3rd edition (WAIS‐III),^17^ and Spatial Span Forward and Spatial Span Backward from the Wechsler Memory Scale, 3rd edition (WMS‐III).^18^ Memory was measured by Logical Memory, Verbal Paired Associates, and Digit Span Backwards from the WMS‐III.^18^ Processing speed was assessed by Symbol Search and Digit Symbol Substitution from the WAIS‐III, Choice Reaction Time, and Inspection Time.^18^, ^19^, ^20^ Crystallized intelligence was measured by the National Adult Reading Test, the Wechsler Test of Adult Reading, and Verbal Fluency (C, F, L).^13^, ^21^

Mini‐Mental State Examination (MMSE) scores were recorded to screen for dementia.^22^ As part of the Scottish Mental Survey 1947, each participant underwent a test of general intelligence at 11 years of age (IQ‐11), the Moray House Test No. 12. The Moray House Test cognitive ability scores have high concurrent validity with gold standard scales of intelligence such as the Stanford–Binet in childhood and the WAIS‐III in older age.^15^, ^16^

### *Carotid Artery Doppler Ultrasound Studies*

Doppler ultrasound imaging of the carotid arteries was performed on an Antares Premium Color Doppler scanner (Siemens, Erlangen, Germany) with 7.5MHz variable frequency probe by experienced neurovascular ultrasonographers. A consultant neuroradiologist cross‐checked all scans. Flow velocity readings were obtained for the common carotid artery (CCA), internal carotid artery (ICA), and external carotid artery. The maximum ICA stenosis was determined based on velocity criteria (ICA peak and end diastolic velocities, and ICA/CCA peak systolic velocity ratio) and caliper measurements at the narrowest point in the ICA or CCA of the residual lumen and diameter of the CCA to calculate the percentage stenosis, and was expressed in the North American Symptomatic Carotid Endarterectomy Trial (NASCET) format.^23^, ^24^ CIMT was calculated using the mean of 3 caliper measurements made on the back wall of the artery over a 1cm‐long segment of the CCA and carotid bulb. From the ICA velocity waveform, we estimated the ICA stiffness by deriving the pulsatility and resistivity indices, as described previously.^25^

### *Brain MRI Acquisition*

All subjects underwent brain MRI at age 73 years on a 1.5T Signa Horizon HDxt clinical scanner (General Electric, Milwaukee, WI). The imaging protocol, described in detail by Wardlaw et al, included T1‐, T2‐, T2*‐, and fluid‐attenuated inversion recovery (FLAIR)‐weighted whole‐brain structural scans.^14^ A high‐resolution whole‐brain 3‐dimensional T1‐weighted volume scan (used here to calculate cerebral cortical thickness) was acquired with a field of view of 256 × 256mm^2^, an acquisition matrix of 192 × 192 (zero‐filled to 256 × 256), and 160 contiguous 1.3mm‐thick slices yielding final voxel dimensions of 1 × 1 × 1.3mm^3^.

### *MRI Processing and Measurement of Cerebral Cortical Thickness and White Matter Hyperintensities*

T1‐weighted images were processed using the automated CIVET pipeline version 1.1.12 (http://www.bic.mni.mcgill.ca/ServicesSoftware/CIVET) developed at the Montreal Neurological Institute. A complete description of the CIVET processing stream has been detailed elsewhere.^26^, ^27^ For each subject, cortical thickness was measured as the distance between 81,924 corresponding points (vertices) of both the GM/white matter and the GM/cerebrospinal fluid interface.^26^ A postprocessing visual quality control protocol was applied to exclude subjects with poor image quality.^27^

FLAIR images were used to calculate each subject's Fazekas score, a standardized visual rating scale that provides a measure of WMH load.^28^ Fazekas scores were assessed by experienced neuroradiologist as periventricular and deep WMHs, each scored from 0 to 3, in each hemisphere.^28^

### *Data and Statistical Analyses*

Local cortical thickness statistical analyses were conducted using SurfStat (www.math.mcgill.ca/keith/surfstat).^29^ All other statistical analyses were performed using SPSS statistical software (SPSS Statistics v23; IBM, Armonk, NY).

The data were analyzed as follows. First, we applied a cross‐sectional design to investigate the association between carotid atherosclerosis markers and vertex‐wide cerebral cortical thickness at age 73 years. Second, we assessed the relationship between carotid atherosclerosis markers and cognitive function at age 73 years and investigated whether any relationship between cognitive function and carotid atherosclerosis was mediated by thinning of the cerebral cortex.

#### *Cross‐Sectional Association between Carotid Atherosclerosis Markers and Cerebral Cortical Thickness at Age 73 Years*

The following carotid atherosclerosis markers were tested: ICA stenosis, CIMT, and ICA velocity, pulsatility, and resistivity indices. These carotid measures were derived from the average (ie, the mean) of the right and left ICA values in each individual. To take into account the severity of carotid disease, the maximum ICA stenosis (observed on the right or the left ICA) was also examined. We used continuous variable measures for the primary analyses to maximize biological validity (as opposed to categorical classifications).

Independent general linear models were applied to examine the association between each carotid measure and vertex‐wide cortical thickness while controlling for gender and age (in days). For the ICA stenosis (0–100%), we tested this association using first‐order linear and quadratic (*F* test) models. As a secondary objective and to facilitate interpretation of results, we divided the study subjects into 4 groups based on the maximum degree of carotid stenosis in the right or left ICA: (1) no carotid stenosis (0%), (2) carotid stenosis between 1 and 24%, (3) carotid stenosis between 25 and 49%, and (4) carotid stenosis ≥ 50%. This categorization was performed to approximately mirror clinical categorization of individuals into those with significant (≥50%, including moderate and severe stenosis according to NASCET criteria) and nonsignificant (<50%, mild stenosis according to NASCET criteria) carotid stenosis. To capture the possible subclinical effect of “nonsignificant” carotid stenosis, we subcategorized individuals with <50% carotid stenosis as described above. Separate general linear models (controlling for age and gender) were applied to examine for between‐group cortical thickness differences.

To determine whether carotid measures have an additional association with cerebral cortical thickness beyond that of measurable VRFs, similar general linear models were repeated introducing the following VRFs as control variables: a history of hypertension, hypercholesterolemia, diabetes mellitus, and smoking; and mean systolic and diastolic blood pressures. History of stroke and/or coronary heart disease, Fazekas scores for WMHs (combined periventricular and deep), MMSE score, and IQ‐11 were also included as covariates in the models. After identifying the strongest associations with carotid stenosis (see Results), we repeated the same model after adding other carotid measures (CIMT, ICA velocity, pulsatility, and resistivity indices) as covariates to determine whether the relationship between carotid stenosis and cortical thickness was independent of other carotid measures. We examined the association between each VRF and cortical thickness independently using separate general linear models after controlling for gender, age, other VRFs, carotid measures, WMHs, MMSE score, and IQ‐11.

To determine whether there was a lateralizing effect to carotid stenosis, we further examined the association between ipsilateral carotid stenosis and cerebral cortical thickness as follows. Subjects with only unilateral carotid stenosis ≥ 25% (left, n = 60; right, n = 74) were selected and compared separately to those with 0% carotid stenosis using general linear models, while controlling for gender, age (in days), VRFs, and the other covariates noted above. Furthermore, subjects with left unilateral carotid stenosis ≥ 25% were compared to those with right unilateral carotid stenosis ≥ 25%.

In each model, a *t* value was calculated for the slope of the β coefficient of the variable of interest at each vertex of the cerebral cortex, producing a 2‐dimensional *t* statistic map. We corrected for multiple comparisons using a false discovery rate (FDR) threshold of *q* ≤ 0.05. As a measure of effect size, we converted *t* statistic maps (using *r* = *t*/√(*df* + *t*2) into partial Pearson correlation coefficients.

#### *The Relationship between Carotid Atherosclerosis Markers, Cortical Thickness, and Cognitive Performance at Age 73 Years*

To determine whether cortical thickness mediates any relationship between markers of carotid atherosclerosis and cognitive performance, we focused on the domain of fluid intelligence due to its documented steeper decline with age relative to crystallized intelligence.^30^ A principal component analysis (PCA) was undertaken to derive a common factor from the following age‐adjusted subsets of the UK WAIS‐III to represent a global measure of fluid intelligence: Matrix Reasoning, Block Design, Digit Span Backward, Letter‐Number Sequencing, Digit Symbol Substitution, and Symbol Search.^17^ These individual measures were highly intercorrelated (Pearson correlation coefficient range = 0.32–0.63). We adopted this approach to increase statistical power and avoid multiple testing.^31^ In a previous investigation of the LBC1936 cohort, fluid intelligence at age 73 years was found to be highly correlated with other cognitive domains, including memory and processing speed (Pearson correlation coefficient range = 0.49–0.76).^31^

After establishing that carotid stenosis has a negative association with cerebral cortical thickness (see first section of Results), a linear regression model was applied to examine the association between carotid stenosis and the PCA‐derived factor from fluid intelligence subtests of the WAIS‐III, before and after adjustment for gender, VRFs, education years, and IQ‐11. Here, standardized coefficients were generated using SPSS after standardization of both the dependent and the independent variables. This was achieved by subtracting each variable's mean from its values, and then dividing the new values by the standard deviation (SD) of each variable. In addition, the association between individual measures of fluid intelligence (see above) and carotid stenosis was examined using independent regression models. Furthermore, an analysis of covariance was applied (controlling for gender, VRFs, and IQ‐11) to test for between‐group differences in fluid intelligence using the same groups of carotid stenosis described above.

**Figure 2:**
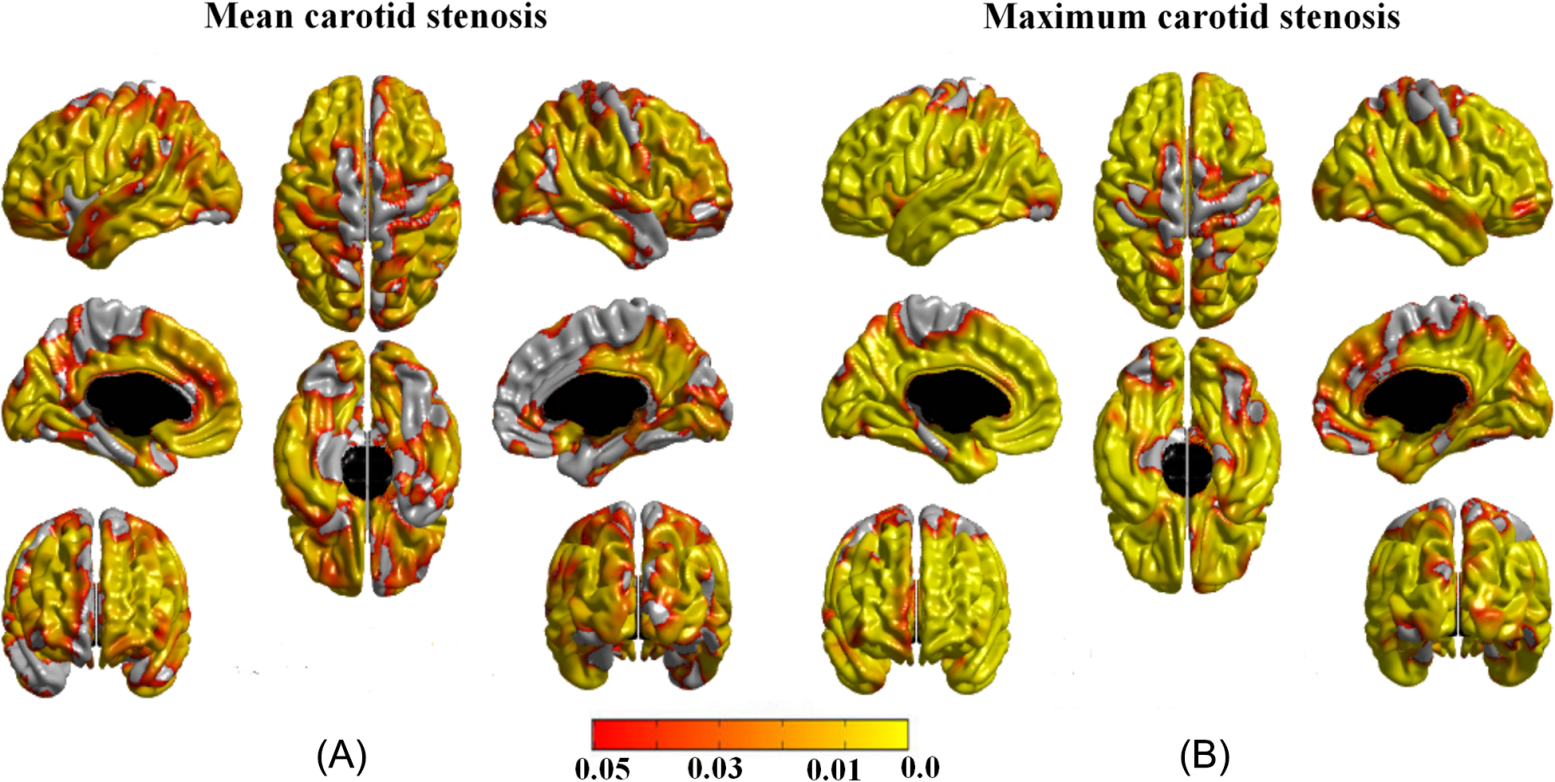
The associations between carotid stenosis and cortical thickness. (A) The association between mean carotid stenosis (average of the left and right internal carotid artery [ICA] stenosis) and cortical thickness, controlling for age (in days) and gender. (B) The association between maximum carotid stenosis (observed on the left or right ICA) and cortical thickness, controlling for age (in days) and gender. The associations in A and B remained significant after controlling for vascular risk factors, intelligence quotient at age 11 years, Mini‐Mental State Examination score, and other carotid measures, affecting both carotid territory and regions supplied by the posterior circulation that are anatomically unrelated to the carotid arteries. Areas in orange–yellow shades represent statistically significant negative associations at false discovery rate (FDR) = 0.05. The color bar represents FDR *q* values.

**Figure 3:**
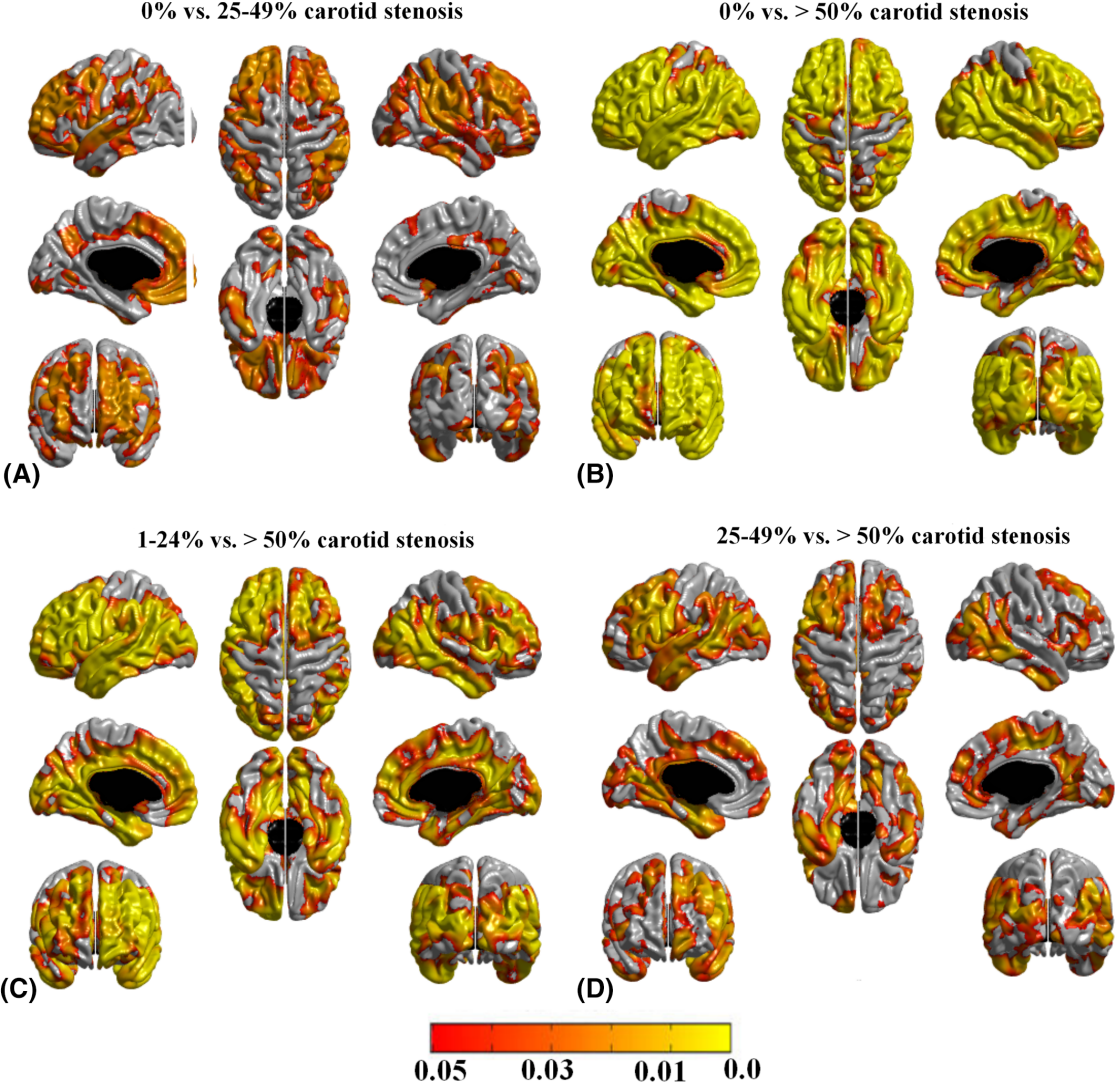
Pattern of cerebral cortical thinning in (A) individuals with 25–49% carotid stenosis compared to those with no carotid stenosis, (B) individuals with ≥ 50% carotid stenosis compared to those with no carotid stenosis, (C) individuals with ≥ 50% carotid stenosis compared to those with 1–24% carotid stenosis, and (D) individuals with ≥ 50% carotid stenosis compared to those with 25–49% carotid stenosis. Age (in days), gender, vascular risk factors, intelligence quotient at age 11 years, Mini‐Mental State Examination score, and other carotid measures were included as covariates. Areas in orange–yellow shades represent statistically significant cortical thinning at a false discovery rate (FDR) = 0.05. The color bar represents FDR *q* values. The group contrasts between 0% and 1–24% carotid stenosis and between 1–24% and 25–49% carotid stenosis did not reach statistical significance.

After establishing that carotid stenosis has a negative relationship with fluid intelligence (see second section of Results), using the PROCESS macro for SPSS, we applied a mediation model to test the hypothesis that the relationship between carotid atherosclerosis and fluid intelligence is mediated by local cortical thickness.^32^ In this model, (1) carotid stenosis was used as carotid atherosclerosis marker, (2) the average thickness of the significant cortical areas derived from the analysis of the relationship between cortical thickness and maximum carotid stenosis was used for the cortical thickness variable (Fig 2B), and (3) the PCA‐derived factor from fluid intelligence subsets of the WAIS‐III was used for the fluid intelligence variable. Using a mediation model, the direct association between carotid stenosis and fluid intelligence was measured after adjustment for the putative mediator variable, the cortical thickness. The indirect effect in the mediation model was assessed using a bias‐corrected bootstrapped confidence interval derived by Monte Carlo sampling.^32^, ^33^ Gender, VRFs, and IQ‐11 were included as covariates in the model.

## Results

### *Association between Carotid Atherosclerosis Markers and Cerebral Cortical Thickness at Age 73 Years*

Controlling for age (in days) and gender, we found no significant relationship between cerebral cortical thickness and CIMT, or with ICA velocity, pulsatility, or resistivity indices. A significant negative association was identified between cerebral cortical thickness and carotid stenosis. This relationship was significant for both mean (average of the right and left ICA) and maximum (observed on the right or the left ICA) carotid stenosis, and it was widespread throughout the cerebral cortex bilaterally, involving the anterior and posterior cortical areas and sparing minor regions of the primary motor and sensory regions after correction for multiple comparisons (see Fig 2). Of note, a trend of a negative association in the primary motor and sensory regions was noted prior to applying FDR corrections. Within areas of significant associations, partial Pearson correlation values ranged between −0.20 and −0.07 (mean ± SD = −0.11 ± 0.02) for mean carotid stenosis, and between −0.22 and −0.07 (mean ± SD = −0.13 ± 0.03) for maximum carotid stenosis. The beta coefficient values ranged between −6.1 × 10^4^ and −0.04 for mean carotid stenosis, and between −7.7 × 10^4^ and −0.04 for maximum carotid stenosis. The first‐order linear model showed more widespread associations than quadratic models and was found to be more efficient (Akaike information criterion was −581.9 for the first‐order model and −567.8 for the quadratic model; Bayesian information criterion was −568.8 for the first‐order model and −554.7 for the quadratic model).

After controlling for VRFs, WMHs, MMSE, and IQ‐11 scores, the above‐noted associations between carotid stenosis and cortical thickness remained significant, with similar effect size (the results are available from the authors on request). Findings were furthermore robust against the addition of other carotid measures (CIMT, and ICA velocity, pulsatility, and resistivity indices) as covariates in the model. Examining the relationship between individual VRFs and cortical thickness revealed significant cortical thinning in individuals who have diabetes relative to those without diabetes and in current smokers relative to non‐ or ex‐smokers.^26^ No significant relationship was identified between cortical thickness, hypertension, hypercholesterolemia, and body mass index after correcting for multiple comparisons.

The categorical carotid stenosis classification variable showed a significant association with cortical thickness in the same anterior and posterior cortical areas. Figure 3 illustrates the results from the secondary group analyses. Compared to the 15% of individuals with 0% carotid stenosis, the 29% of individuals with 25 to 49% and the 4% of individuals with ≥ 50% carotid stenosis displayed widespread bilateral cerebral cortical thinning. As expected, the anatomical distribution of the cortical thinning was more extended in the small proportion of individuals with ≥50% carotid stenosis. A similar pattern of thinner cortex was also noted in individuals with ≥50% carotid stenosis relative to those with 1 to 24% carotid stenosis (see Fig 3). The effect is seen in both carotid artery and posterior circulation territories bilaterally. The group contrasts between 0% and 1 to 24% carotid stenosis and between 1 to 24% and 25 to 49% carotid stenosis did not reach statistical significance.

**Figure 4:**
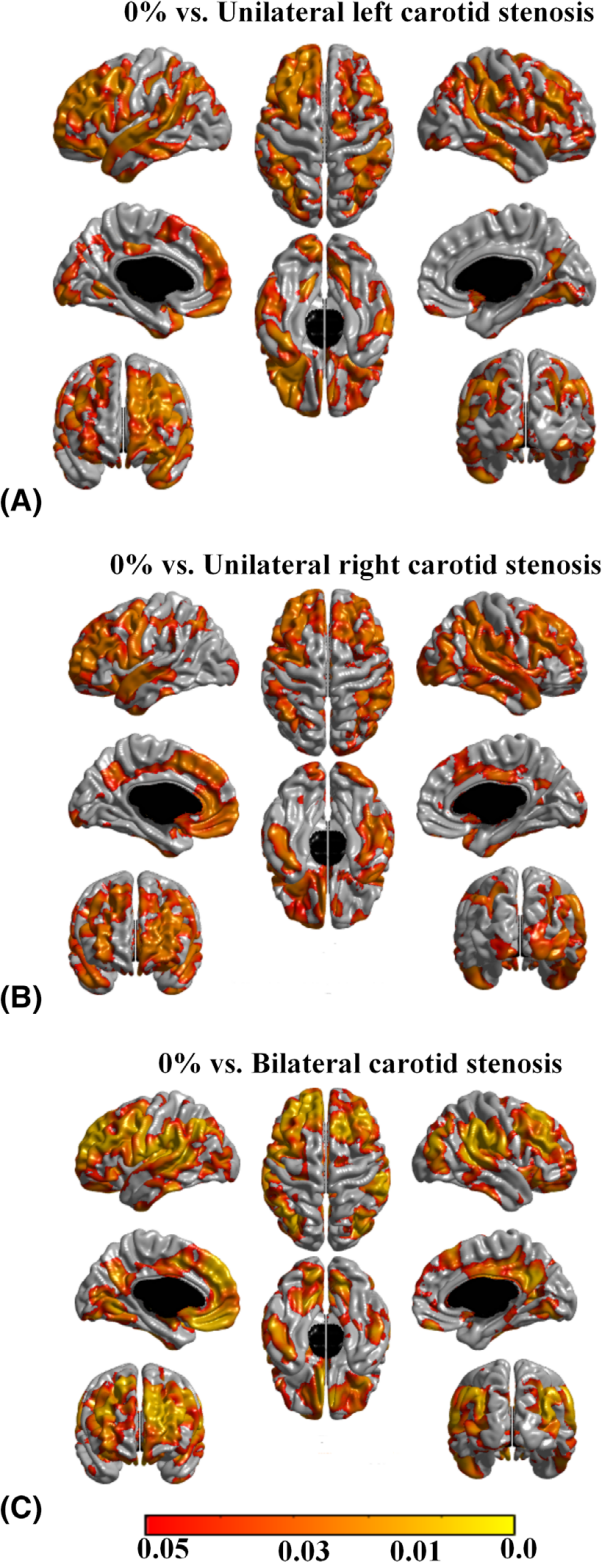
Pattern of cerebral cortical thickness reduction in (A) individuals with ≥ 25% left carotid stenosis compared to those with no carotid stenosis, (B) individuals with ≥ 25% right carotid stenosis compared to those with no carotid stenosis, and (C) individuals with ≥ 25% bilateral carotid stenosis compared to those with no carotid stenosis. Areas in orange–yellow shades represent statistically significant cortical thinning at a false discovery rate (FDR) = 0.05. The color bar represents FDR *q* values.

When individuals with only unilateral carotid stenosis ≥25% were compared to those with 0% carotid stenosis, significant cortical thinning was noted bilaterally in prefrontal, parietal, and lateral temporal cortices, without evidence of lateralization (Fig 4A, B). A similar pattern of cortical thinning was noted in individuals with bilateral carotid stenosis ≥ 25% (see Fig 4C). When subjects with left unilateral carotid stenosis ≥ 25% were compared to those with right unilateral carotid stenosis ≥ 25%, no significant group differences were noted.

### *Relationship between Carotid Atherosclerosis Markers, Cortical Thickness, and Cognitive Performance at Age 73 Years*

Linear regression modeling confirmed a negative association, as found in our previous analysis,^12^ between carotid stenosis and fluid intelligence at age 73 years (maximum carotid stenosis: standardized beta coefficient = −0.151, *p* = 0.001; mean carotid stenosis: standardized beta coefficient = −0.131, *p* = 0.003; Table 2). Controlling for sex, VRFs, education years, and IQ‐11 made only minor differences to the strength of these associations. Examining the association between the abovementioned individual measures of fluid intelligence and carotid stenosis using independent regression models revealed significant negative associations between carotid stenosis and the following WAIS‐III subsets: Block Design, Digit Span Backward, Digit Symbol Substitution, and Symbol Search.

**Table 2:** Relationship between Carotid Stenosis and Fluid Intelligence at Age 73 Years^a^

Carotid Measure at Age 73 Years	Adjusted for Gender, VRFs, Years of Education, and IQ‐11	Fluid Intelligence at Age 73 Years Standardized Beta	*p*
Mean carotid stenosis	No	−0.131	0.003
	Yes	−0.101	0.007
Maximum carotid stenosis	No	−0.151	0.001
	Yes	−0.108	0.001

a Before and after adjustment for gender, VRFs, education years, and IQ‐11.

IQ‐11 = intelligence quotient at 11 years; VRF = vascular risk factor.

**Figure 5:**
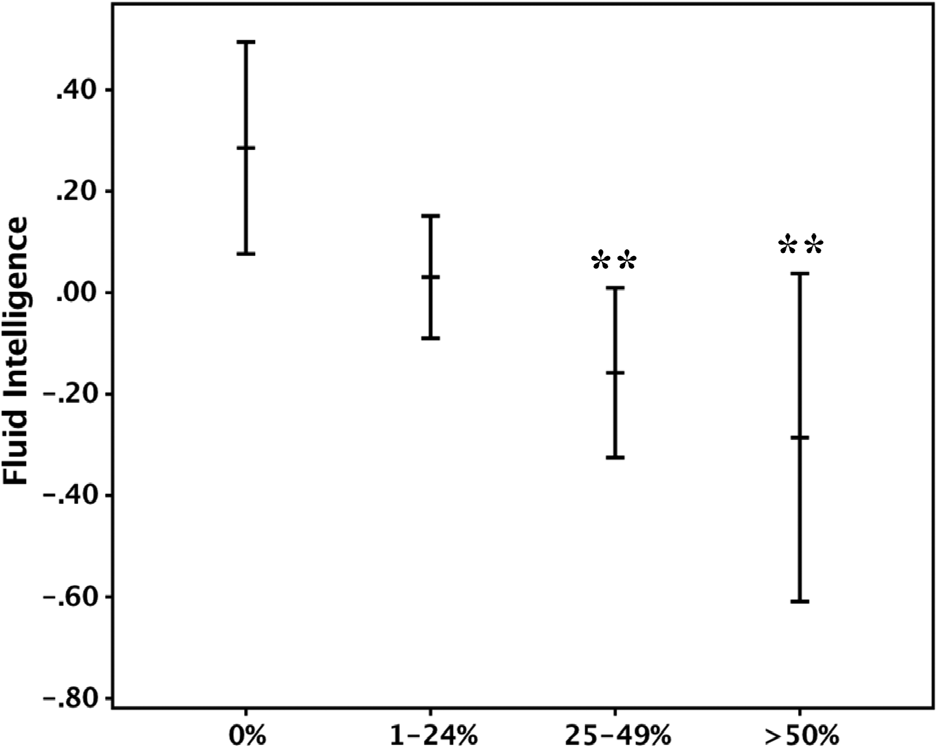
Mean fluid intelligence score in individuals with 0%, 1–24%, 25–49%, and ≥50% carotid stenosis. Error bands represent 95% confidence intervals. The score was derived using a principal component analysis from the following subsets of the Wechsler Adult Intelligence Scale, 3rd UK edition (WAIS‐III): Matrix Reasoning, Block Design, Digit Span Backward, Letter‐Number Sequencing, Digit Symbol Substitution, and Symbol Search. **p < 0.01 (comparisons were made against individuals with 0% carotid stenosis). Covariates: age, gender, vascular risk factors, and intelligence quotient at age 11 years.

The group analyses revealed that individuals with 25 to 49% (Cohen *d* = 0.46, *p* = 0.002) and ≥50% carotid stenosis (Cohen *d* = 0.65, *p* = 0.009) had lower fluid intelligence score relative to those without carotid stenosis (Fig 5). No statistically significant score difference was noted between individuals with 0% and 1 to 24% carotid stenosis.

**Figure 6:**
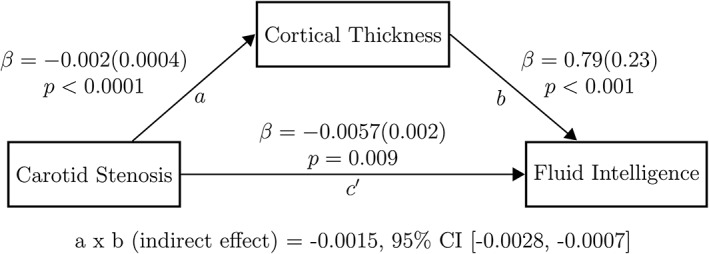
The result of the mediation model applied to examine the effect of cortical thickness on the relationship between carotid stenosis and fluid intelligence is displayed. To investigate the association between carotid atherosclerosis, cortical thickness, and fluid intelligence, (1) maximum carotid stenosis was used as a marker for carotid atherosclerosis; (2) the average thickness of the significant cortical areas derived from the analysis of the relationship between cortical thickness and carotid stenosis was used for the cortical thickness variable (shown in Fig 2B); and (3) the principal component analysis–derived general component from the following subsets of the Wechsler Adult Intelligence Scale, 3rd edition was used for the fluid intelligence variable: Matrix Reasoning, Block Design, Digit Span Backward, Letter‐Number Sequencing, Digit Symbol Substitution, and Symbol Search. Each arrow is directed from a predictor variable to the outcome variable: (1) the relationship between carotid stenosis and cortical thickness is denoted by path *a*; (2) the relationship between cortical thickness and fluid intelligence, adjusted for carotid stenosis, is denoted by path *b*; (3) the direct effect of carotid stenosis upon fluid intelligence is denoted by path *c′*, and was measured after adjustment for cortical thickness, gender, intelligence quotient at age 11 years, and all vascular risk factors. Beta coefficients and related *p* values displayed adjacent to each path represent unstandardized regression coefficients (standard errors) generated (an ordinary least squares regression model) as part of the mediation model using the PROCESS macro for SPSS. A formal test of the significance of the mediation effect was carried out, for which the results are listed at the bottom of the figure. The indirect effect in the mediation model (the degree to which the effect of carotid stenosis on fluid intelligence is believed to be transmitted through mean cortical thickness) was assessed using a bias‐corrected bootstrapped confidence interval (CI) derived by Monte Carlo sampling. Number of bootstrap samples = 10,000.

Using the mediation model, the negative relationship between carotid stenosis and mean cortical thickness (unstandardized beta coefficient = −0.002, standard error [SE] = 0.0004, *p* < 0.00001) and the relationship between fluid intelligence and mean cortical thickness (unstandardized beta coefficient = 0.790, SE = 0.23, *p* = 0.0008) were significant (paths *a* and *b*, respectively in Fig 6). Furthermore, a negative association between carotid stenosis and fluid intelligence, prior to adjustment of the indirect effect through mean cortical thickness, was noted (unstandardized beta coefficient = −0.0072, SE = 0.002, *p* = 0.0009). The mediation effect was statistically significant (unstandardized beta coefficient = −0.0015, 95% confidence interval = −0.0028 to −0.0007), and indicates that the association between carotid stenosis and fluid intelligence was partly mediated by the carotid stenosis–related thinning of the cerebral cortex. Approximately 22% of the effect of carotid stenosis on fluid intelligence was mediated by cortical thickness. The direct effect of carotid stenosis upon fluid intelligence remained significant after adjustment for cortical thickness (unstandardized beta coefficient = −0.0057, SE = 0.002, *p* = 0.009; path *c* in Fig 6).

## Discussion

This study identified a significant widespread cross‐sectional association between carotid stenosis and whole‐brain cortical thickness in community‐dwelling older individuals at age 73 years. A negative relationship following a linear monotonic model was demonstrated between carotid stenosis and cortical thickness independent of other carotid measures, VRFs, WMHs, and IQ‐11. As in our prior analysis,^12^ we confirmed a significant negative association between measures of fluid intelligence and carotid stenosis at age 73 years. Having previously excluded any mediation by WMHs,^12^ our novel finding is that this association was partly mediated by carotid stenosis–related thinning of cerebral cortex. A further significant and novel finding is that the carotid stenosis–cortical thickness association was present in all vascular territories, including the regions supplied by the carotid and vertebrobasilar arteries, irrespective of the side of carotid stenosis, with no lateralizing difference in cortical thickness in subjects with a unilateral carotid stenosis. This negative association did not appear restricted to individuals with clinically significant carotid stenosis, but increased in an almost dose–response relationship from mild to severe degrees of carotid stenosis, across both the anterior and posterior circulation territories, appearing least strong in the posterior frontal and anterior parietal cortical regions. This weak association between carotid stenosis and primary motor and sensory cortical thickness could be related to image processing and cortical thickness measurement limitation at the brain's vertex or lack of adequate statistical power to detect a smaller effect in these brain regions.

The mechanism underlying the association between carotid stenosis and cortical thickness remains to be determined. Compromised blood flow secondary to hypoperfusion has previously been suggested as a possible underlying mechanism for brain atrophy associated with carotid atherosclerosis.^34^, ^35^ However, only 4% of our cohort had ≥50% carotid stenosis, which may result in notable hemodynamic and flow compromise, making this hypothesis less likely to explain our observations of widespread cortical thinning even at a lower degree of carotid stenosis (ie, in individuals with ≥25% carotid stenosis). This, along with other findings in this work, indicates that carotid stenosis is unlikely to affect the thickness of the cerebral cortex through a direct hemodynamic effect. Instead, it would appear that the carotid stenosis represents a proxy marker for vascular alterations within the cerebrum, including the possible cumulative effect of both measurable and nonmeasurable VRFs, or other global exposure (eg, smoking, inflammatory mediators, or other difficult to measure variable), which also results in thinner cerebral cortex throughout the brain's anterior and posterior circulation territories.^26^, ^36^, ^37^ To this effect, we assessed whether smoking was the culprit. Further explorations confirmed that the relationship we noted between carotid stenosis and cortical thickness persisted despite factoring in pack‐years of smoking, and also in individuals with no history of smoking (the results are available from the authors on request). We know from previous analysis that the relationship between cortical thickness at age 73 years and cognitive ability at age 70 years is mostly explained by IQ‐11.^38^ However, there was little change in the associations we noted between carotid stenosis and cortical thickness after adjusting for IQ‐11.

Previous investigations that explored the association between markers of carotid atherosclerosis and cognitive function have reported conflicting results.^8^, ^9^, ^10^, ^11^ A decline in executive functioning and processing speed was reported previously using a cross‐sectional design in nondemented older adults and middle‐aged individuals with increased CIMT.^10^ Similarly, the Framingham study reported poorer performance on executive functioning tasks in individuals with carotid stenosis ≥ 50%.^5^ However, using a 6‐year longitudinal design, a study of the LBC1936 with a larger sample than the one included in the current analyses (it was not restricted to subjects with available MRI) did not report an association between changes in cognitive function and mean (average) carotid stenosis after controlling for VRFs and IQ‐11.^12^ Conversely, parameters of carotid wall stiffness (including pulsatility and resistivity indices) were found to associate with slower processing speed and worse visuospatial function at age 70 years, in addition to declining crystallized intelligence from age 70 to 76 years.^12^ In the current study, we adopted an independent strategy to analyze the cognitive data compared to Wardlaw et al.^12^ We focused on examining the relationship between a PCA‐derived measure of fluid intelligence and carotid stenosis at age 73 years due to the previously documented steeper decline of fluid intelligence with age.^30^ By using a continuous variable measure of maximum (compared to mean) carotid stenosis, we were able to demonstrate a negative association between a composite score of fluid intelligence and the maximum carotid stenosis at age 73 years. Our findings provide further evidence toward possible mechanistic explanations by demonstrating that this negative association is partly mediated by the negative relationship between carotid stenosis and cerebral cortical thickness.

Given that carotid stenosis here likely represents a proxy marker for another vascular alteration within the cerebrum affecting both the anterior and posterior circulation, we extracted the mean thickness of all the cerebral cortex regions that showed a negative association with carotid stenosis, and used this measure as a representation of carotid stenosis–related cortical thinning. After we adjusted for VRFs and WMHs, our mediation model suggested that cortical thickness mediates approximately 22% of the relationship between carotid stenosis and fluid intelligence.

This study has some limitations. First, there were a small number of individuals with significant carotid stenosis (≥50%), limiting our ability to explore the association between carotid stenosis, whole‐brain cortical thickness, and cognitive function with high‐degree carotid stenosis. Second, only about two‐thirds of the available carotid measures were used in this analysis, as a large proportion of T1‐weighted brain MRI data were not available, reducing the overall sample. Third, whereas we accounted for the effect of small‐vessel disease by factoring in the load of WMHs and history of strokes, other possibly relevant markers, including microbleeds, were not measured. Furthermore, as guided by the main findings of cortical thickness analyses, we focused on investigating the relationship between carotid stenosis and fluid intelligence. The relationship of other carotid measures (eg, CIMT) with fluid intelligence was not explored. Finally, although we provided hypotheses on possible processes that underlie the relationship between carotid stenosis and cortical thickness, we did not formally evaluate cortical thickness differences in cerebral cortical regions supplied by the different vascular territories. The exact mechanisms, therefore, remain unknown, and many of the findings point to a corelated global process that results in carotid atheroma, reduced cortical thickness, and impaired cognition.

In conclusion, our findings demonstrate that carotid stenosis is cross‐sectionally related to thinner cerebral cortex and lower fluid cognitive abilities at age 73 years. The negative association between carotid stenosis and cortical thickness appeared to, in part, mediate the association between carotid stenosis and decline in fluid intelligence. The presence of this relationship in individuals with asymptomatic mild carotid atheroma suggests that carotid stenosis could be a marker for accelerated cortical aging with a negative impact on cognition, and therefore strengthens the case for efforts to improve population vascular health throughout adulthood as part of a strategy to reduce aging‐related cognitive decline.

## Author Contributions

S.A., S.K., T.‐V.N., A.Th., J.M.W., I.J.D., and S.D. contributed to the conception and design of the study. S.A., S.K., T.‐V.N., B.C.B, S.R.C, J.C., A.Ta., A.C.E, J.M.S, M.E.B., J.M.W., and I.J.D. contributed to the acquisition and analysis of data. S.A., J.M.W., I.J.D., and S.D. contributed to drafting the text and preparing the figures.

## Potential Conflicts of Interest

Nothing to report.
